# Supplementary material for: Antidiabetic activities of Bolanthus spergulifolius (Caryophyllaceae) extracts on insulin-resistant 3T3-L1 adipocytes
Source: PLoS One. 2021 Jun 16;16(6):e0252707. doi: 10.1371/journal.pone.0252707 (PMC8208533; doi:10.1371/journal.pone.0252707)
Supplement: S1 Raw image — (DOCX) [file pone.0252707.s001.docx]

**S1 Raw İmage**

**Glut-4 protein levels in 3T3-L1 cells by Western-blot**

- Cell lysates were in SDS-PAGE to proteins by size. Proteins were trabsfered onto a PVDF membrane via Mini Trans Blot Cell Module (Bio-Rad, USA). The membrane was blocked in 5% Skimmed Milk and TBS-Tween.


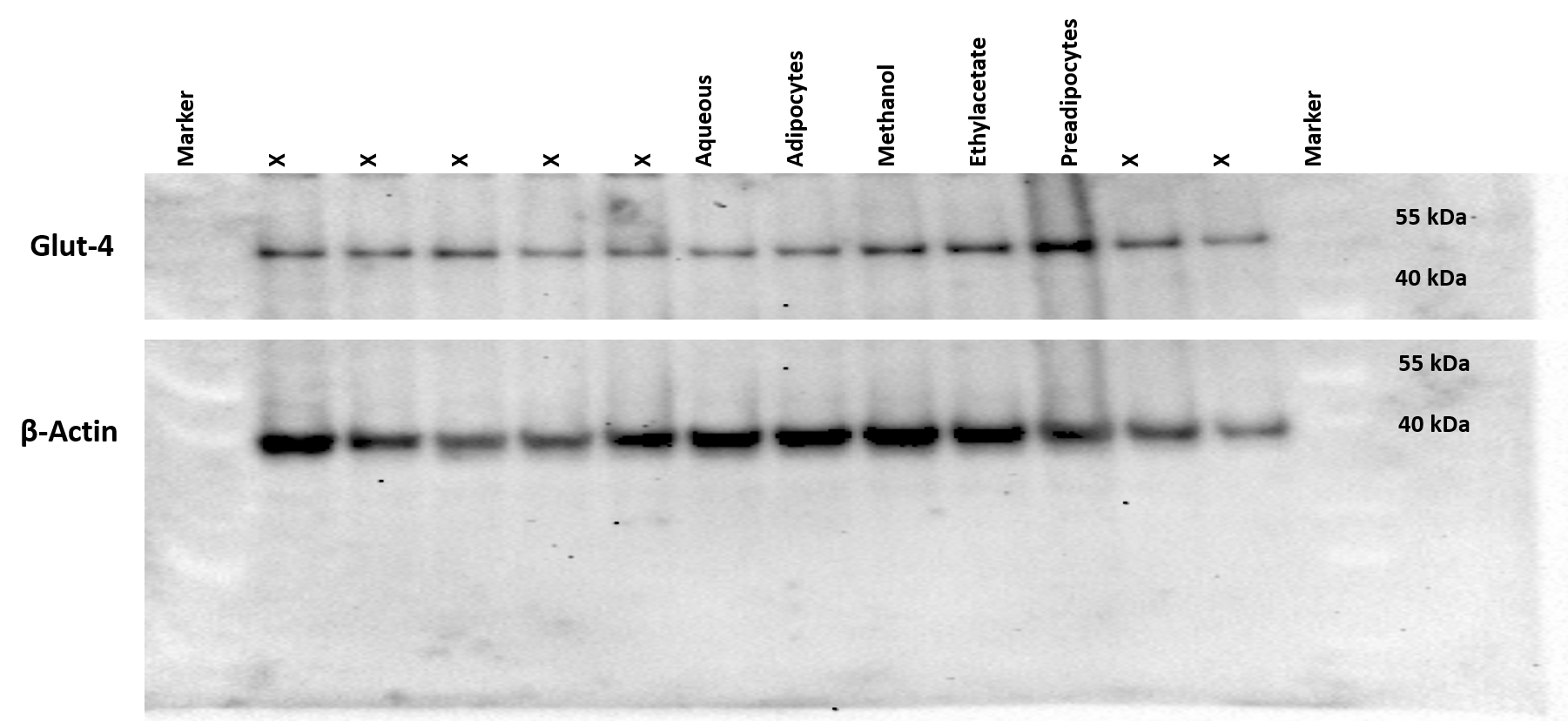


- Figüre upper part: As primary antibody Anti-Glut-4 (1/100; ab358; Abcam, Cambrige, UK) in 5% skimmed milk at overnight at +4°C was used. As secondary antibody goat Anti-mouse IgG (1/1000; ab205719; Abcam, Cambrige, UK) with horse radish peroxidase (HRP) for 60 min. at 37 °C was used.
- Figüre lower part: As primary antibody Anti-β-Actin (1/100; ab8226; Abcam, Cambrige, UK) in 5% skimmed milk at overnight at +4°C was used. As secondary antibody goat Anti-mouse IgG (1/1000; ab205719; Abcam, Cambrige, UK) with horse radish peroxidase (HRP) for 60 min. at 37 °C was used.
- For HRP detection ECL Western Blotting reagent (Bio-Rad, Hercules, USA) was used.
